# Observations on the Good Effects of Caustics in Cases of White Swellings of the Joints

**Published:** 1793

**Authors:** Bryan Crowther

**Affiliations:** Surgeon to Bridewell and Bethlem Hospitals.


### XIV. *Obfervations on the good Effects of Cauftics in Cafes of White Swellings of the Joints. By Mr.* Bryan Crowther, *Surgeon to Bridewell and Bethlem Hofpitals.*

THE great fuccefs generally attending the ufe of cauftics in curvatures of the fpine, arifing from a difeafed ftate of the vertebræ, or of their ligaments, has induced me to extend the remedy to white fwellings of the joints of the limbs. I am aware that Mr. Pott applied Cauftics a little above and a little below fcrophulous joints of the the limbs *, but his practice was not ſo ſucceſsful as in caſes of curved ſpine. The failure has been ſuppoſed to be owing to the greater diſtance of the limb from the heart; it may be rather apprehended, that in the extremities the application was not near enough to the diſeaſe.

This opinion has ſomething more than probable conjecture to recommend itſelf; as in the laſt of the caſes I ſhall relate, I made one iſſue on the integuments covering the external condyle of the thigh-bone, and another in the hollow above the knee; and repeated attentive obſervations and enquiries ſatisfied me that the external iſſue afforded greater relief than the one made above the joint, which induced me to bring the iſſue from above the knee, by the plaſtered ſponge, down upon the inner condyle of the thigh-bone; and the advantage ariſing from merely changing the ſituation of the ſore has fully anſwered my expectations.

Theſe ideas of the malady and the remedy led me to embrace the firſt opportunity of applying the cauſtic on each ſide of the diſeaſed joint. In doing this I was induced to apply it

* Vid. Vol. iii. of Mr. Earle's Edition of Mr. Pott's works, pages 497 and 498.

where where the ſoft parts were thickeſt. When the eſchars were ſeparated I kept the ſores open, by an occaſional dreſſing of the *Ung. Cantharid.* or by eſcharotic applications; but before it could be known what effect the cauſtic would produce, I found that the iſſue ointment, as well as the eſcharotic applications, gave more pain than the patient could well bear. To promote a diſcharge from the ſores in an eaſier way, therefore, I made uſe of a piece of ſponge dipt in melted wax, and preſſed flat, which was cut nearly to the ſize of the ſore, to which it was confined by croſs ſlips of adheſive plaſter. The ſame intentions may be anſwered by pouring melted wax on two or three layers of lint laid on each other; but whether the lint or the ſponge be uſed, the waxed application ſhould be leſs than the ſores, ſo as to allow room ſufficient for the granulations at their edges to riſe above its ſurface; thus will the waxed lint or ſponge be confined in its ſituation, the uſe of the bliſter ointment be rendered unneceſſary, and the bandage required to keep on the dreſſings will prevent any fungus that can be diſtreſſing to the patient, or troubleſome to the ſurgeon. Should the ſores contract, they may be enlarged to the extent wiſhed for, by applying ſponge that has been been dipped in the melted empl. attrahens. Sponge thus prepared will ſwell out, which is not the caſe when it is filled merely with wax.

The patients, in fine weather, ſhould be in the open air as much as poſſible, aſſiſted by a crutch to take off the weight of the body from the diſeaſed limb; they ſhould be allowed a nouriſhing diet, with the uſe of bark, and, in ſome caſes, opium.

That the propoſed means of relief do not confine thoſe who labour under this diſeaſe to the houſe, is a very happy circumſtance to all, but more eſpecially to hoſpital patients.

Of the uſual remedies in this complaint, bliſters ſeem to be the moſt promiſing. They afford indeed, in general, only a temporary relief; but they ſhould be applied to the joint when the pain is excruciating, as they prepare the ſkin for the action of the cauſtic; it being certain that the latter does the moſt execution in caſes where the ſkin has been recently bliſtered.

This happened in the caſe of Stephen Raſhfield *; in that inſtance we were obliged, on

* See Caſe I.

account of the pain the patient felt, to examine the dreſſings; and, on removing them, found that the cauſtic immediately covering the new-form'd ſkin, had there made a deeper eſchar than on the original ſkin. In ſcrophulous joints, not attended with much pain, as generally is the caſe when the diſeaſe is confined to bone, recourſe ſhould be immediately had to the cauſtic.

When bliſtering diſtreſſes the patient by ſtrangury, perhaps ſinapiſms might be ſubſtituted with equal effect. I have obſerved that they will procure more diſcharge; and this from a ſurface leſs ſuperficial than a bliſter. In a manuſcript, in my poſſeſſion, without a date, entitled 'Commentaries on Monro's Oſteology' I find ſinapiſms recommended in dropſies of the joints, in preference to bliſters, as being more manageable, and their effect more laſting.

The following caſe related by Fabricius ab Aquapendente *, from its analogy to our preſent ſubject, will probably not be thought unworthy of peruſal. "Nobilis vir in conſiſtenti "ætate conſtitutus, cum ex fluxione pituitoſa "& frigida genu haberet valde tumidum, et ita "obduratum, ut penitus immobile factum eſſet,

* De chirurg. operat. cap. 106.

"et inflexibile, vocati ad hujufmodi curam

"Capivaccius, et ego, exiftimavimus cafum effe

"infanabilem; tamen ut aliquid tentaremus, fo-

"latii potius egrotantis gratia, cœpimus ipfum

"purgare, ut tuta adminiftraremus, interea a

"quodam empirico emplaftrum ex quadam

"herba confectum, quam ego puto flammu-

"lam fuiffe, impofitum fuit, quod ftatim in-

"flammationem infignem in genu excitavit,

"cum rubore, calore & dolore; et ab ea hora

"cepit æger aliquantulum genu movere; fen-

"fimque ita proceffit, ut tandem fanus factus

"fit."

This difeafe is now and then cured, though more frequently only relieved, by an abfcefs forming in the vicinity of a difeafed joint. In thefe cafes the fuppurative procefs feems neceffary to a cure; but when fymptoms of recovery do not fpeedily follow, the patient becomes hectic, and at length dies, after enduring great pain and mifery, unlefs this fatal termination is prevented by a timely amputation of the limb.

The cauftic I have made ufe of in this difeafe has been the cauftic powder prepared at Apothecaries Hall, and made into a pafte by the addition of a little *Lixiv. Saponac.* and foft foap; but but whatever efcharotic may be preferred it fhould remain as long a time on the part as from the thicknefs of the integuments can be done with fafety; or, rather, till a fufficient depth of efchar is made, that can fecure to the furgeon a granulating furface. The pain the patient feels, and the introduction of the point of a lancet into the efchar, will beft enable us to afcertain the execution done by the cauftic.

The fize of the fores muft be determined by the patient's health, and the progrefs the difeafe has made.

As the bones at the articulations are more thinly covered than other parts of the body, I would propofe that the fores in general fhould be made as large as a crown piece. How long they fhould be kept difcharging can be determined only by their effect; but I would recommend their remaining open (though a little contracted) for fome time after the cure.

It is worthy of remark, that in the cafes where the cauftic, with the fubfequent treatment as above recommended, has been tried, collections of difeafed fluids in and about the fcrophulous joint have been difperfed, and thereby the morbid enlargement of the joint reduced.

### CASE I.

The first case in which the caustics were applied was that of Stephen Rashfield. He had been afflicted, according to his own account, with a white swelling of his knee eight years. During the first four of these the disease, he observed, had made a considerable progress, till an abscess formed on the outside of the joint, which was opened by Mr. Pitts, late surgeon of St. Bartholomew's Hospital, and discharged half a pint of matter. This abscess remained open ten months, during which time he experienced great relief. Soon after this period he relapsed, and from that time became worse.

He applied to me in April 1792; and, besides this scrophulous knee, which was greatly enlarged and much contracted, he had nodes of the tibia on the other leg, attended with nocturnal pains, which he said could not be venereal, as he had never been in the way of contracting such a complaint. They yielded, however, to a mercurial course, which afforded no relief to the diseased joint. His pains being excruciating, a blister was applied over the whole knee; this this procured a temporary eaſe, but did not tend to alter the ſtate of the joint. Cauſtics were therefore tried in the manner I have recommended, and though this caſe did not ſucceed, yet the patient for many weeks found himſelf relieved by them, and the ſize of the knee was reduced.

In the month of December he was admitted into St. Thomas's Hoſpital, where he underwent amputation.

### CASE II.

Charles Sellers applied to me in June 1792, on account of a ſcrophulous knee which had been diſeaſed eighteen years. It was nearly twice as large as the ſound knee, and was attended with violent pain. A bliſter was applied to it, which afforded him no relief; cauſtics therefore were had recourſe to, and when the eſchars were digeſted off he became eaſier; and in a few weeks the knee was free from pain, and much diminiſhed in ſize. His health at the ſame time was greatly improved.

Some of my medical friends, who had ſeen him previouſly to the application of the cauſ-

tics, noticed their effects with pleaſure. He continued to go on well, and we had every reaſon to hope for a gradual cure till the middle of November, when a large abſceſs formed in the ham. After this his health ſuffered ſo much from the quantity of matter diſcharged, that he requeſted me to take off his limb, which I did on the 25th of December. The diſeaſed knee was then reduced very nearly to the ſize of the other. His ſtump is now perfectly healed, and his health re-eſtabliſhed.

### CASE III.

Elizabeth Platt, of Hoxton, about ſeven years of age, a little before Chriſtmas 1791, was obſerved by her mother to walk lame, which was attributed to chilblains; but, on undreſſing her, her mother diſcovered that one knee was larger than the other. On the 6th of April following I viſited the patient, and found her labouring under ſymptoms of hectic fever. Her knee joint was then much contracted, incapable of flexion or extenſion, and much enlarged, particularly the inner condyle of the thigh bone. As her pain was not violent, except when when the limb was moved, recourſe was immediately had to the cauſtic, which has effected a re-eſtabliſhment of her health, a diminution in a great degree of the enlargement of the joint, with a perfect recovery of its motion, and a conſiderable relaxation of the tendons of the flexor muſcles, the remaining contraction of which ſeems the only impediment to the full exertion of the limb; as ſhe can bend the joint with eaſe, and can extend the leg as far as the contraction of the tendons will admit of.

Large diſcharges ſuddenly produced from the ſurface of the iſſues ſeem to relieve the diſeaſe, and amend the conſtitution, much more than a regular and gradual drain. This remark was made in Platt's caſe ſince Chriſtmas laſt; as before that time the means of dilating the ſores by a plaſtered ſponge, and conſequently increaſing the diſcharge, had not occurred to me.

It may be thought, and with good reaſon, that my experience of the efficacy of cauſtics in theſe caſes is too inconſiderable to prove what degree of confidence they may merit as a remedy in ſuch complaints. The good effects that were produced by them in the inſtances I have related ſeem indeed ſufficient to recommend them

 to to the notice of furgeons in fimilar cafes; but the utility of this, or of any other mode of treatment can be fatisfactorily afcertained only by an extenfive trial; and I fubmit the above obfervations to candid practitioners that they may augment or correct them.

*Bofwell Court,*

April 27th, 1793.

